# The successful use of a bespoke OssDsign cranial plate to reconstruct an occipital defect following excision of a recurrent epithelioid sarcoma

**DOI:** 10.1016/j.jpra.2020.01.002

**Published:** 2025-02-25

**Authors:** Oliver Bloom, Naveen Goddard, Vasileios Yannoulias, Simon Eccles

**Affiliations:** The Craniofacial Unit, Department of Plastic Surgery, Chelsea and Westminster Hospital, 369 Fulham Road, London SW10 9NH, United Kingdom

**Keywords:** Cranioplasty, Bespoke, Reconstruction, Epithelioid sarcoma

## Abstract

A cranioplasty has a number of known associated complications including infection for non-biological implants and bone flap resorption where autologous grafts are used. In recent years, bioactive ceramic cranial implants have been developed as a new reconstructive option. The OssDsign cranial plate (OssDsign AB, Uppsala, Sweden) was first introduced in 2010 and consists of an interconnected mosaic of hydroxyapatite tiles mounted onto a 3D-printed titanium mesh. Each tile is composed of a monetite, beta-calcium pyrophosphate, beta-tricalcium phosphate and brushite compound designed to mimic the process of coupled bone formation once implanted.

This case presents a patient's journey from diagnosis of an epithelioid sarcoma over the posterior scalp and its management in the following 7 years. Initial excision of the lesion was reconstructed with a tissue expander and local rotational flap. Recurrence of the disease 3 years later was treated with a course of radical radiotherapy. Persistent osteomyelitis over the next 3 years resulted in chronic ulceration and exposed bone in the treated area. As the first part of a 3-stage treatment plan, two separate tissue expanders became infected. The multidisciplinary team therefore chose to use a bespoke OssDsign cranial plate combined with a deep inferior epigastric perforator (DIEP) free flap to provide a definitive single operative solution. The advantages over other reconstruction options include that the plate can be removed should further excision be required, greater potential for long-term integration with surrounding tissues and the ability to be soaked in antibiotic to reduce the risk of infection.

## Case report

This case presents a 37-year-old female patient who originally presented with skin changes over her occiput in 2010. A biopsy identified this as an epithelioid sarcoma, a rare soft tissue tumour that has a known propensity for local recurrence and metastasis.^1^ She underwent excision and reconstruction with a local rotational flap following tissue expansion using an implantable expander. In 2013, she had a recurrence of the disease, which was treated with radical radiotherapy. She subsequently developed osteomyelitis in 2015 and this persisted until August 2016. Following CT and MRI scans and with multidisciplinary team (MDT) discussion in early 2017, it was decided that she should have a three-stage reconstruction of the 10 cm x 7 cm defect of her skull. This would involve a combination of tissue expansion and a custom-made PEEK or hydroxyapatite-coated titanium implant. However, she developed further infection on both occasions, so tissue expanders were inserted in January and May 2017. The management was therefore revised to a single resection and immediate reconstruction operation was performed using a custom hydroxyapatite-coated titanium cranioplasty implant from OssDsign and deep inferior epigastric perforator (DIEP) free tissue coverage.

Once the team decided on the OssDsign cranial plate, several meetings were held in which 3D models and CT images were used to plan the reconstruction. With the design finalised, the titanium mesh and pre-mould were printed and finished. The manufacturing process involved 3D printing of the defect perimeter, moulding, setting and drying of the implant followed by material analysis. From the acceptance of the design proposal, the cranioplasty implant was delivered within 23 days.

**Figure 1:**
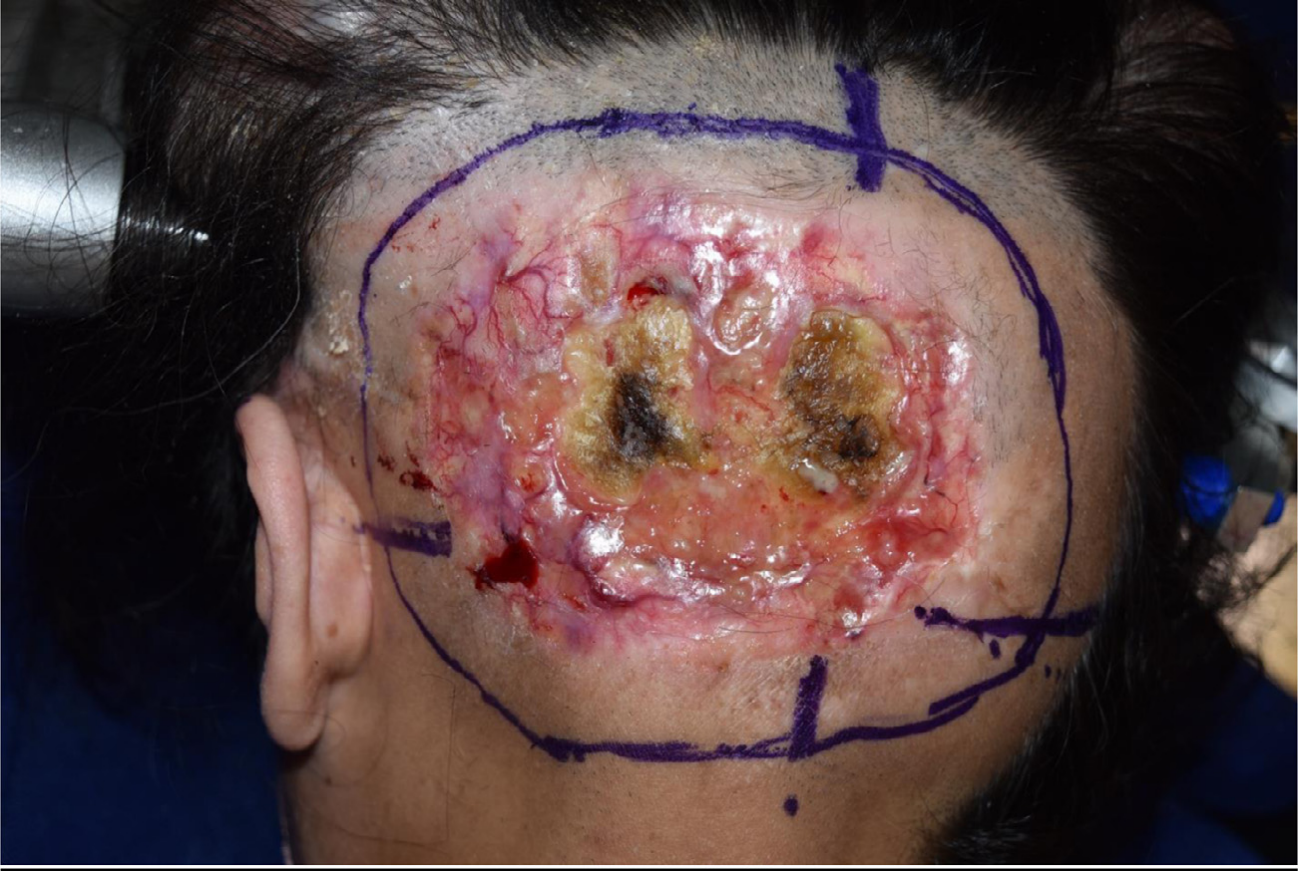
The skin marking of the lesion.

**Figure 2:**
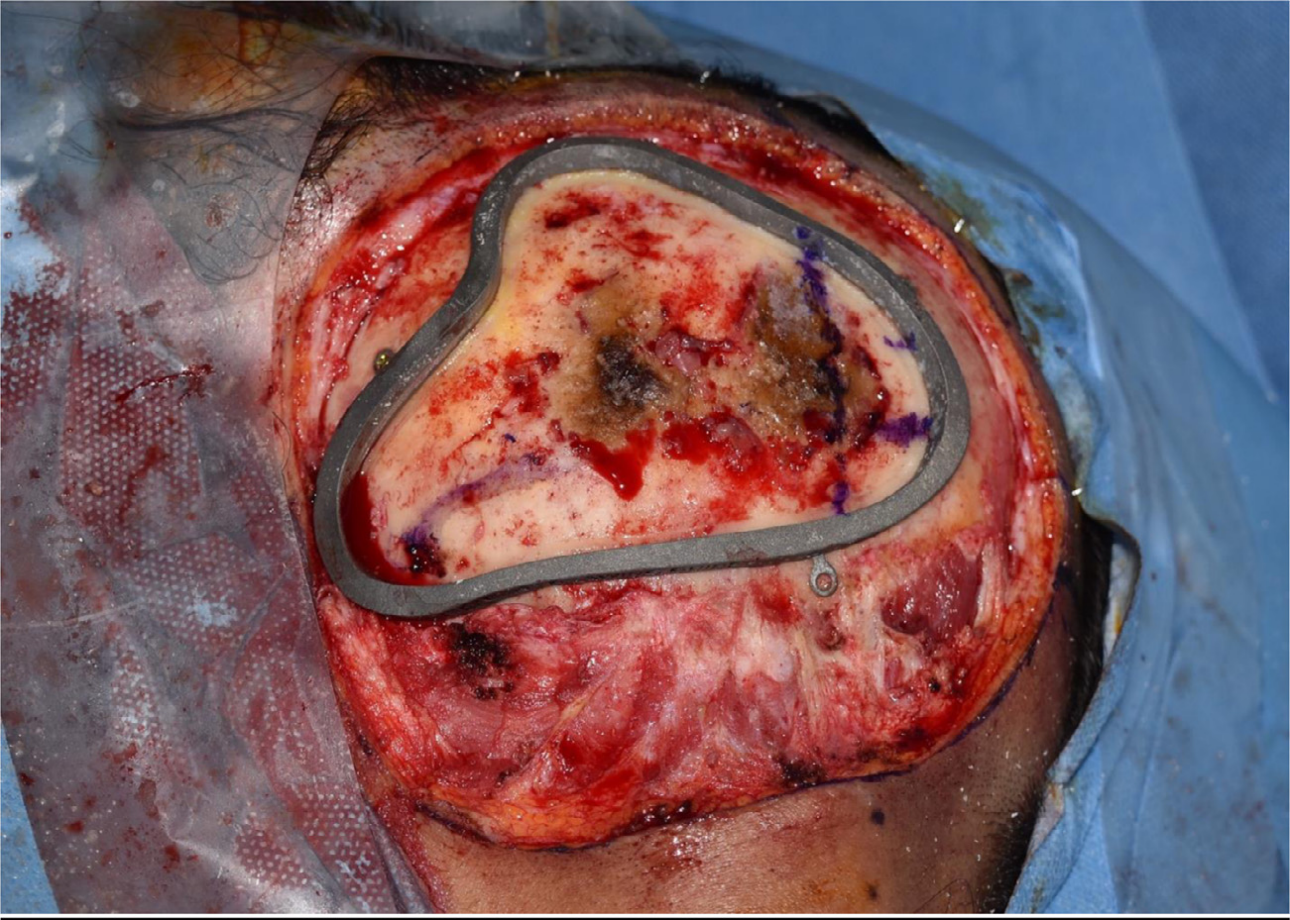
The bony lesion with the cutting guide in situ and the dural venous sinuses marked.

During the surgical procedure, the cutting guide area was marked on the scalp and the estimated location of the transverse and sigmoid dural venous sinuses noted (Figure 1). This complex area of surgical anatomy was mapped accurately by the production of a model of the occipital area of the skull and a cutting template. An occipital craniotomy was performed using the custom-made OssDsign cutting guide, which measured 8 cm x 7 cm. Care was taken to preserve the venous sinuses (Figure 2).

**Figure 3:**
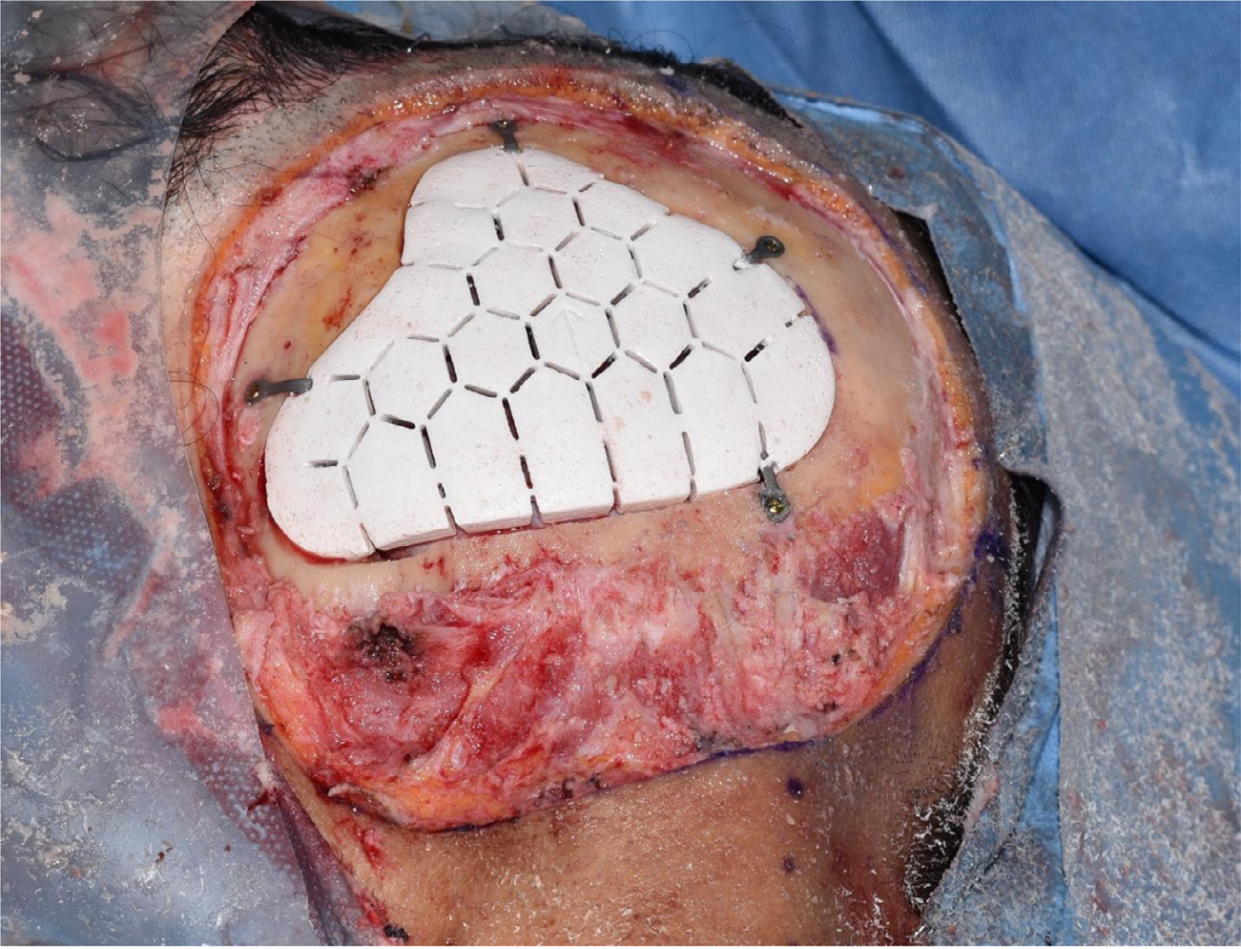
The secured OssDsign implant in the defect.

**Figure 4:**
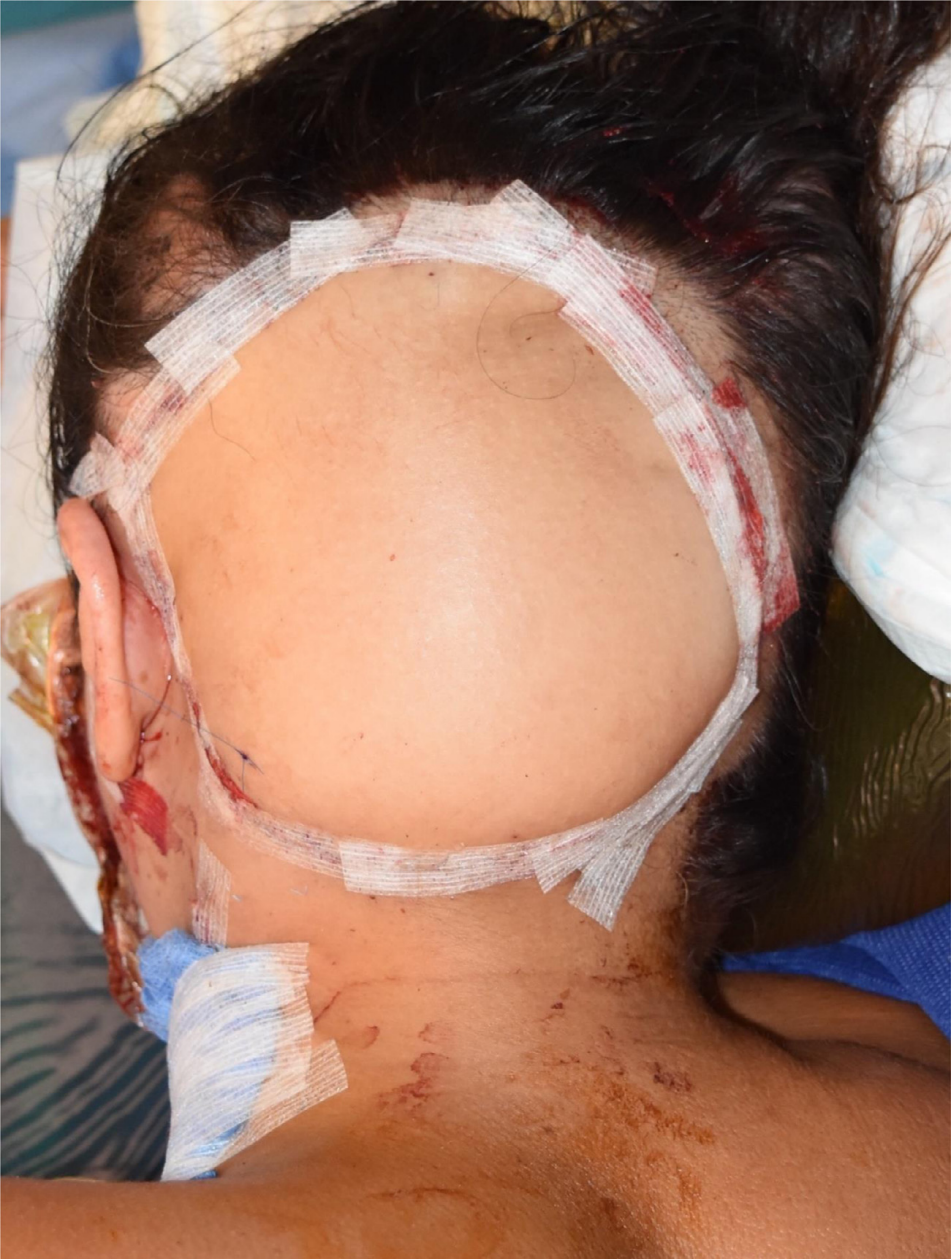
The appearance of the flap at the end of the operation.

A sample of dura was taken and sent for histological analysis to see if the disease had spread deep to the bone. Because of the accuracy of the 3D printed design, only slight adjustments were required. The implant was inserted and secured with screws after being soaked in a solution of Vancomycin for 10 min (Figure 3). The DIEP flap was raised, the left temporal region explored and the superficial temporal artery and veins chosen for the anastomosis of the DIEP flap. The flap was then inset into the scalp defect (Figure 4).

The post-operative course for the patient was uneventful, and there were no wound-healing issues in the immediate post-operative period. Since the surgery, the flap has shrunken down slightly and has been covered by a hairpiece. This was aesthetically acceptable for the patient and she continues to be followed up by the plastic surgery and oncology departments.

## Discussion

Several techniques have been employed to reconstruct cranial defects, including the use of autologous bone and synthetic substitutes such as PEEK and titanium implants. Both PEEK and titanium plates are proven to give excellent functional results, with PEEK implants having the advantage of being more anatomically compatible to the specific cranial defect.^2^ In recent years, bioactive ceramic cranial implants have been developed, including those comprising of calcium phosphate. Their interconnected mosaic structure using ceramic tiles provides not only a permanent reconstructive solution but also one that can stimulate bone healing in the affected area to fully incorporate the implant. Such implants have been shown to provide a restored cranial vault without complications, for patients who have previously undergone unsuccessful reconstructive surgeries.^3^, ^4^, ^5^

The third generation of the OssDsign Cranial PSI was first introduced in 2014, and consists of a 3D titanium mesh with a ceramic coating consisting of monetite, beta-calcium pyrophosphate (BPPi), beta-tricalcium phosphate and brushite. The composition is designed to mimic the process of coupled bone formation once implanted. Gene expression analysis and histology have confirmed the presence of osteoclast activity as well as new bone formation on the mosaic tiles of the OssDsign plate.^4^ This suggests that cell-mediated resorption of BPPi occurs. It is thought that the addition of BPPi may prolong the time for resorption, therefore allowing sufficient time for coupled bone formation to occur.^3^ In addition, the mosaic design of the plate, with 1 mm spaces in between each tile, allows the implant to be adjusted perioperatively and provides enhanced circulation of tissue fluid for optimal tissue integration. Furthermore, PET and CT scans have confirmed bone formation in the central part of the implant. The distal location of this area from host bone suggests that each tile possesses its own osteoinductive properties.

There were a number of key criteria that were used by the MDT to choose an appropriate implant. The OssDsign cranial plate still provided the excellent structural properties and customisable 3D structure afforded by PEEK implants, but had a number of added advantages. A custom cutting guide was produced alongside the implant, which facilitated the exact excision of all the diseased bone while keeping the size of the defect to an absolute minimum. Hydroxyapatite-coated implants have some key benefits over the alternative choices.^6^ When compared to PEEK implants, they display better biomimetic properties with much greater integration with the surrounding tissues.^7^ Furthermore, recent studies have demonstrated a low explantation rate (1 in 42 cases).^5^ In this case, the patient's excised bone and soft tissue were seen to have strands of the original neoplasm at the deep margin. Therefore, if the cancer were to reoccur, the easy removal of the implant would allow for further reconstruction to take place. It can also be impregnated with antibiotic solution to further reduce the risk of infection.

The bespoke OssDsign plate provided an effective material for a cranioplasty in this case, building upon its previous use in traumatic reconstruction.^3^ The success of this implant has led to the introduction of an equivalent OssDsign facial implant with potential applications in reconstructing facial bone after trauma, tumour resection or growth retardation due to irradiation.

## Conclusion

This case describes the long-term sequelae stemming from the treatment of a recurrent epithelioid sarcoma that led to a state of chronic inflammation, wound breakdown and poor healing on the occipital aspect of the patient's scalp. The OssDsign Cranial PSI combined with a DIEP free flap provided an effective solution to the patient's lesion, which has the flexibility to be raised should further deep debridement be required.

After years of daily wound dressings and frequent hospital visits, this innovative method has facilitated a single operative reconstruction, saving multiple trips to the theatre and many days in hospital for the patient.

## Declaration of Competing Interest

None of the authors have any financial or personal relationships with other people or organisations that could inappropriately influence or bias their work.
